# Diet quality during pregnancy, adolescent brain morphology, and cognitive performance in a population-based cohort

**DOI:** 10.1016/j.ajcnut.2024.08.018

**Published:** 2024-10-08

**Authors:** Yuchan Mou, Pauline W Jansen, Hong Sun, Tonya White, Trudy Voortman

**Affiliations:** 1 Department of Epidemiology, Erasmus MC, University Medical Center Rotterdam, The Netherlands; 2 The Generation R Study Group, Erasmus MC, University Medical Center Rotterdam, The Netherlands; 3 Department of Child and Adolescent Psychiatry/Psychology, Erasmus MC, University Medical Center Rotterdam, The Netherlands; 4 Department of Psychology, Education and Child Studies, Erasmus University Rotterdam, The Netherlands; 5 Department of Radiology and Nuclear Medicine, Erasmus MC, University Medical Center Rotterdam, The Netherlands; 6 Meta-Research Innovation Center at Stanford (METRICS), Stanford University, Stanford, CA, United States

**Keywords:** dietary patterns, MRI, brain volumes, surface area, gyrification, cortical thickness, intelligence quotient, prospective cohort study, adolescents

## Abstract

**Background:**

Diet quality during pregnancy may affect offspring’s neurobiology and cognitive performance in childhood. However, little is known about underlying mechanisms and potential long-term effects.

**Objectives:**

To examine associations of diet quality during pregnancy with offspring pre- and early-adolescent brain morphology and to investigate whether brain morphology mediates associations of diet quality during pregnancy with full-scale intelligence quotient (IQ) in early adolescence.

**Methods:**

We studied 2223 and 1582 mother–child dyads with brain scans collected using magnetic resonance imaging at ages 10 and 14 y in the population-based Generation R Study in The Netherlands. We assessed dietary intake during pregnancy with 293-item food-frequency questionnaires and calculated predefined diet quality scores (total score 0–15), reflecting adherence to dietary guidelines. Cognitive performance was assessed using Wechsler Intelligence Scale for Children-V at age 14 y. We examined associations using multiple regression models, corrected for multiple testing.

**Results:**

After adjustment for child age, sex, socioeconomic factors, maternal age, smoking, and psychopathological symptoms during pregnancy, we found that higher diet quality during pregnancy was associated with a larger total brain (B: 4.54, 95% confidence interval [CI]: 1.80, 7.28), cerebral white matter (1.83, 95% CI: 0.56, 3.10), cerebral gray matter (1.99, 95% CI: 0.63, 3.35), and subcortical volumes (0.16, 95% CI: 0.04, 0.28) of children at age 10 y. Similar results were found for age 14 y. Widespread differences in cortical thickness, gyrification, and surface area in both hemispheres were also observed. Better diet quality during pregnancy was associated with higher full-scale IQ scores of adolescents, particularly on verbal comprehension and matrix reasoning. The associations between diet quality during pregnancy and full-scale IQ in early adolescence were partially mediated by brain volumetric markers in pre-adolescence.

**Conclusions:**

Diet quality during pregnancy was associated with structural brain alterations in the offspring, which partly explained the relation between prenatal dietary patterns and cognitive outcomes in children.

## Introduction

The human brain undergoes rapid volumetric growth in the conception period [1], accompanied by the maturation of brain function and cognitive complexity throughout childhood [2]. This ongoing and substantial development of brain morphology and function depends on an adequate supply of nutrients to support the high energy demands [3]. Nutrition during pregnancy is particularly important in shaping the foundation for offspring’s cognitive development, given that the most rapid growth and highest plasticity of the brain occurs during the first 1000 d of life [4].

Accumulating evidence underscores the importance of maternal dietary intake during pregnancy, for example deficiencies of certain nutrients can compromise neurodevelopment during critical periods of brain development and alter gene expression through epigenetic modification of chromatin in the brain, which can result in structural changes that persist into adulthood [[5], [6], [7]]. Complementary to the research focus on individual nutrients, recent epidemiological research investigated the associations of overall dietary patterns during pregnancy with child neurodevelopment [8]. The perspective of examining overall dietary intake is currently emphasized by dietary recommendations and is useful as dietary components synergistically interact with each other in such a way that can influence human health [9]. Indeed, a meta-analysis summarizing evidence from 18 observational studies suggested that a better diet quality during pregnancy has a small positive association with neurodevelopment in early childhood [10], with more consistent findings within cognitive domains [[11], [12], [13], [14], [15], [16], [17], [18]], although the majority of the studies used fish intake as a marker for diet quality and only 1 study has focused on overall diet quality [11]. Recent prospective cohort studies have extended the evidence by showing long-term associations between overall dietary patterns during pregnancy and cognitive outcomes in mid-childhood [19,20]. For example, unhealthy dietary patterns during pregnancy have been linked to lower intelligence quotients (IQ) in children aged 8 y [19], and a better-quality diet during pregnancy has been associated with higher intelligence and better executive functioning skills in children at a median age of 7.7 y [20]. However, the neurobiological underpinnings of these effects of diet during pregnancy on children’s cognitive function remain unclear.

Structural brain alterations have been suggested to be a detectable neurobiological marker on the pathway of maternal nutrition to child cognitive development. Rodent studies have demonstrated that a high-fat diet during the gestation period induces neuroanatomical changes in the mesolimbic pathway [21], medial prefrontal cortex [22], and dendrites of hippocampal and amygdala [23] as well as extensive structural changes throughout the brain [24] in the offspring into adulthood, all of which were linked to reduced cognitive development [25,26]. Studies using primate models have shown that nutrient restriction during pregnancy compromised widespread structural cerebral development [27] and subsequently altered cognitive and behavioral performance in the offspring [28,29]. Although less is known about the relationship between diet during pregnancy and brain morphology of offspring in humans, we have recently shown a mediating role of brain morphology in the relation of diet in early- and mid-childhood with cognitive performance in adolescence [30]. Additionally, differences in volumetric measures of brain morphology have been linked to cognitive phenotypes in children [[31], [32], [33]].

The purpose of this study was to investigate the associations between diet quality during pregnancy and offspring’s brain morphology in pre- and early adolescence in a large population-based cohort, and to investigate whether diet quality-related differences in brain morphology mediated associations of diet quality during pregnancy with full-scale IQ in early adolescence. We assessed diet quality by using a predefined diet quality index, reflecting the degree of adherence to dietary recommendations. In addition, as maternal diet might act as a marker of child diet, we investigated whether associations of maternal diet were independent of offspring diet in childhood.

## Methods

### Study design and population

This study was embedded in the Generation R Study, a population-based prospective cohort from fetal life until young adulthood which is designed to identify early environmental and genetic determinants of growth, development, and health during the life course. Participants eligible for this study were pregnant individuals with a delivery date from April 2002 through January 2006 living in Rotterdam, The Netherlands [33]. The study was approved by the Medical Ethics Committee of Erasmus Medical Center, Rotterdam. Written informed consent was obtained from all participating children and their parents.

Of the 7893 mother–child pairs who consented for the postnatal follow-up, 6485 mothers provided valid dietary information during pregnancy, collected from 2002 through 2006. Brain images of children were obtained when children were 10 y (collected from January 2013 through November 2015) and 14 y of age (collected from October 2016 through December 2018). We excluded children who did not visit the neuroimaging research center or did not provide consent for the MRI scan at either of these ages. Furthermore, children were excluded if images could not be reconstructed, had poor quality, if major incidental findings were found, or if the gyrification index could not be calculated. The final study population therefore comprised 2223 children for analysis of brain morphology at age 10 y, 1582 children for analysis of brain morphology at age 14 y, and 872 children with brain morphology at both ages (Supplemental Figure 1).

### Measures

#### Diet quality during pregnancy

Mothers’ dietary intake during pregnancy was measured using a semiquantitative 293-item food-frequency questionnaire (FFQ) in the first trimester of gestation (median 13.6 wk, IQR: 12.4, 16.2). Energy and food intake were calculated using the Dutch food composition table from 2006 [34]. The FFQ was validated against three 24-h recalls among 71 pregnant individuals living in Rotterdam. Intra-class correlation coefficients for macronutrient intakes ranged from 0.5 to 0.7 [35]. A predefined diet quality score for pregnant individuals was developed reflecting adherence to Dutch dietary guidelines [36]. The diet quality score includes the following 15 food components and cut-offs: vegetables (≥200 g/d), fruit (≥200 g/d), whole grains (≥90 g/d), legumes (≥135 g/w), nuts (≥15 g/d), dairy (≥300 g/d), fish (≥100 g/w), tea (≥450 g/d), grain quality (ratio whole grains of total grains), soft fats and oils (ratio of total fat), red meat (≤375 g/w), sugar-containing beverages (≤150 g/d), alcohol (yes/no), salt (≤6 g/d), and folic acid supplement use in early pregnancy (used periconceptionally/in the first 10 wk/no). For each component, the ratio of the reported intake and the recommended intake was calculated, except for alcohol and folic acid supplements. For example, a woman with a fruit intake of 120 g/d received a score of 0.6 (120 g/d divided by a recommended 200 g/d) for the fruit component. For any component intake exceeding the recommended intake, the score was truncated to 1. The scores of sugar-containing beverages, red meat and salt were reversely coded, with higher scores on these food components reflecting lower intake. Alcohol intake and folic acid supplements use were scored as follows: no alcohol intake scored 1 and any alcohol intake scored 0; periconceptional intake of folic acid supplement scored 1, in the first 10 wk of gestation scored 0.5, and no intake in these periods scored 0. The total diet quality score was calculated by summing the individual food component scores, resulting in an overall score ranging from 0 to 15, with a higher score representing a healthier diet. We evaluated the diet quality score against nutrient intake and found positive associations of the score with intake of favorable nutrients (for example, dietary fiber) and negative associations with intakes of unfavorable nutrients (for example, saturated fat), suggesting desirable internal validity of the diet quality score (Supplemental Table 1).

#### Brain morphometry

At the age of 10 y (median 9.9, IQR 9.7, 10.1) and the age of 14 y (median 13.9, IQR 13.6, 14.3), the children were invited to the MRI center. Before the MRI procedure, the children participated in a mock MRI session. MR images were acquired on a 3.0 Tesla GE Discovery MR750w MRI system (General Electric Healthcare) scanner using an 8-channel head coil. The high-resolution T1-weighted sequence was obtained using a 3-dimensional coronal inversion recovery fast spoiled gradient recalled (IR-FSPGR, BRAVO) sequence. The overview of the sequences and imaging protocol was reported in detail elsewhere [37]. MRI scans were evaluated by trained researchers and neuroradiologists using a predefined protocol for incidental findings, and a detailed list of incidental findings presented in the Generation R Study population is reported elsewhere [38]. Briefly, children who had incidental findings and were referred to a pediatric neurologist for clinical imaging and follow-up were defined as having major incidental findings, which includes primary brain tumors. Children with the presence of major incidental findings were excluded from the analyses to avoid introducing variability in brain morphology related to major underlying health issues that likely unrelated to diet quality during pregnancy.

Volumetric segmentation and cortical reconstruction were processed using FreeSurfer version 6.0 analysis suite (https://surfer.nmr.mgh.harvard.edu/) with standard processing procedure [39]. Volumes of the right and left hemispheres were summed to obtain global and subcortical volumes, including total brain, cerebral white and gray matter, and subcortical volumes. For surface-based morphometry, cortical thickness, surface area, and gyrification were quantified. The quality of cortical reconstructions was visually inspected, and images were removed in case of insufficient quality [39].

#### Cognitive performance

Estimated full-scale IQ of children was derived from a subset of the Wechsler Intelligence Scale for Children-Fifth Edition (WISC-V) assessed when they were 13–16 y of age. The WISC-V is an instrument assessing individual cognitive functioning in 6- to 16-y olds. In collaboration with Pearson (Pearson Clinical Assessment), 4 core subtests were selected from the WISC-V to derive an estimated full-scale IQ. The 4 subtests included vocabulary, matrix reasoning, digit span, and coding, which measure verbal comprehension, fluid reasoning, working memory, and processing speed, respectively. All 4 subtests were administered by trained research assistants. The detailed method of administering the 4 subtests is described elsewhere [40]. Raw subsets scores were first converted to age-standardized t-scores (ranging from 1 to 19) based on Dutch norm scores and were summed and converted to an estimated full-scale IQ.

#### Covariates

We selected several potential confounders that are associated with both exposure and outcome based on previous research [[41], [42], [43], [44]] and used a directed acyclic graph to decide the minimal list of confounders to include in the main analysis. Information on child sex was collected from medical records filled in by obstetricians and community midwives. Self-reported questionnaires during pregnancy were used to collect information on household income, maternal national origin, education, psychopathology symptoms, and on smoking. Energy intake during pregnancy was estimated using the FFQs. Net household income was categorized into <1200€, 1200–2200€ and >2200€ per mo. National origin of mothers was determined based on their country of birth and grouped into Dutch and non-Dutch. Mothers’ highest education attainment was dichotomized into low (from no education up to lower vocational training) and high (higher vocational training/university). Maternal psychopathology symptoms were measured by calculating the global severity index, using the Brief Symptom Inventory [45] assessed at the third trimester of gestation. Smoking during pregnancy was categorized as never, until pregnancy was known, and continued. We considered child diet quality at the age of 8 y as a potential mediator in the association and therefore we included this in a separate model. This was measured by an FFQ, from which a predefined diet quality score including 10 food components (total score ranging from 0 to 10) was quantified reflecting adherence to age-specific dietary recommendations from Dutch dietary guidelines, as described in detail elsewhere [41]. Information on duration of exclusive breastfeeding was obtained from postnatal questionnaire at 2, 6, and 12 mo after birth, and we categorized it into 3 groups: <2 mo, ≥2∼<6 mo, and ≥6 mo.

### Statistical analysis

Sample characteristics were described as mean and SD for continuous variables with normal distributions, median and IQR for continuous variables with skewed distributions, or percentages for categorical variables.

We investigated the relation between diet quality of the mothers during pregnancy and brain morphology of their children (total brain, cerebral white and gray matter, subcortical volumes, cortical thickness, surface area, and gyrification) using multiple linear regression models per brain volume assessment at ages 10 and 14 separately. We used 2 models to examine the associations. In model 1 (main analyses), we adjusted for child sex and age at neuroimaging assessment, household income, maternal age, education, national origin, smoking during pregnancy, psychopathological symptoms during pregnancy, and energy intake. In model 2, we additionally adjusted for child diet quality at 8 y of age. In the analyses of global and subcortical brain morphometry, correction for multiple testing was performed using the Benjamini–Hochberg approach [46] for 4 primary outcomes per age, that is 8 tests in total, with a false discovery rate of 0.05. In vertex-wise analyses of surface-based morphometry, the Gaussian Monte Carlo Simulations with a cluster-wise correction were used to correct for multiple testing. The cluster-forming threshold was set to *P* = 0.001, which corresponds to a false-positive rate of 0.05 [47]. Bonferroni corrections were further applied for each brain hemisphere (*P* < 0.025 cluster-wise).

To investigate the mediating role of brain morphology collected at 10 y of age in the association of diet quality with full-scale IQ collected at age 14 y, we performed causal mediation analyses for those diet quality-brain volume associations that remained significant after multiple testing correction. For each mediation analysis, we specified mediator models for the conditional distribution of the diet-related brain morphology given the diet quality and covariates, and outcome models for the conditional distribution of the full-scale IQ given the diet quality, diet-related brain morphology, and covariates. Models were adjusted for the same set of covariates as in the aforementioned models. Each mediation analysis was analyzed with 1000 simulations using the quasi-Bayesian Monte Carlo method on normal approximation to obtain the estimates of the average direct, indirect, and total effects.

To mitigate potential bias arising from missing values in covariates, we employed multiple imputations under the assumption that data were missing at random [48], generating 10 datasets through 50 iterations using the MICE package within R. Causal mediation analyses were conducted using the “mediation” package in R, and the combined results are presented. Surface-based morphometry analyses utilized the QDECR package [49] in R version 3.6.3, whereas all other statistical analyses were performed using R version 4.0.3 (R Foundation for Statistical Computing). Statistical significance was set at a 2-sided α level of <0.05.

We conducted several sensitivity analyses to test the robustness of results. First, information on maternal and child characteristics between respondents and non-respondents with FFQ and neuroimaging data at age 10 and 14 y was compared individually. Second, we repeated multiple regression analyses in mothers with a Dutch and non-Dutch national origin separately. Third, we repeated the analyses and additionally adjusted for breastfeeding duration in the models. Fourth, to take into account children’s brain growth trajectories, we used linear mixed model analyses to test whether diet quality during pregnancy is associated with changes in global brain volumes over time. Fifth, to examine whether the differences observed in global brain volumes were proportionally different in relation to head size or the result of regional volume expansion of certain regions of brain, we additionally adjusted for intracranial volume in the models. Sixth, to examine whether the observed associations were driven by folate levels, we adjusted for maternal folate concentrations during pregnancy measured from venous blood in early gestation (mean = 13.3 wk, SD = 1.9 wk) in the models. Last, to assess whether any of the 15 food components used to construct the diet quality score were driving the associations, we re-ran the main analysis on the associations of brain volumes and IQ with diet quality score excluding each of the 15 food components one at a time and additionally adjusting for the excluded component in the models.

## Results

The characteristics of the study population are reported in Table 1. In general, the characteristics for the different brain imaging samples (at ages 10 and 14 y) were similar. Mothers were on average 31.2 y (SD 4.6) old at enrollment, and most of them had high educational levels and a Dutch national origin. The average diet quality score during pregnancy was 7.8 (SD 1.6) out of the total score of 15, and the average diet quality score for children at age 8 y was 4.5 (SD 1.2) out of the total score of 10. The correlation between maternal diet quality during pregnancy and child diet quality at age 8 y was 0.29. Information on missing values of covariates is reported in Supplemental Table 2.

**TABLE 1:** Maternal and child characteristics of included mother–child pairs in the Generation R Study^1^.

	Sample with data at 10 y *n* = 2223	Sample with data at 14 y *n* = 1582
Maternal characteristics		
Age at enrollment	31.2 (4.6)	31.2 (4.7)
Educational level (high), *N* (%)	1458 (65.6%)	1011 (63.9%)
Household income per month, *N* (%)		
<1200 €	267 (12.0%)	201 (12.7%)
1200–2200 €	489 (22.0%)	388 (24.5%)
>2200 €	1467 (66.0%)	995 (62.9%)
National origin, *N* (%)		
Dutch	1420 (63.9%)	975 (61.6%)
Non-Dutch	803 (36.1%)	607 (38.2%)
Smoking during pregnancy, *N* (%)		
Never	1745 (78.5%)	1223 (77.3%)
Until pregnancy was known	205 (9.2%)	127 (8%)
Continued	273 (12.3%)	233 (14.7%)
Diet quality score (range: 0–15)	7.8 (1.6)	7.7 (1.6)
Child characteristics		
Age at the neuroimaging assessment, median (IQR), (y)	9.9 (9.8, 10.3)	13.9 (13.6, 14.3)
Sex (girls), *N* (%)	1123 (50.5%)	850 (53.7%)
Diet quality scores at age 8 y (range: 0–10)	4.5 (1.2)	4.5 (1.2)
Full-scale IQ score	103.7 (13.5)	103.1 (13.6)
Vocabulary score, t score	10.0 (2.9)	9.9 (3.0)
Matrix reasoning score, t score	9.5 (2.7)	9.4 (2.6)
Digital span score, t score	9.8 (2.7)	9.6 (2.8)
Coding score, t score	13.0 (3.3)	12.9 (3.3)

Abbreviations: IQ, intelligence quotient; IQR, interquartile range.

1 Values are mean (SD) for continuous variables with a normal distribution, medians (IQR) for continuous variables with a skewed distribution, or valid numbers (%) for categorical variables. Missing data of covariates were imputed with multiple imputation (*m* = 10 imputations, the information of missing values of covariates is in Supplemental Table 2).

### Associations of diet quality during pregnancy with brain morphology

Table 2 reports that better diet quality during pregnancy was associated with larger total (B: 4.54, 95% CI: 1.80, 7.28), cerebral white matter (B: 1.83, 95% CI: 0.56, 3.10), and cerebral gray matter (B: 1.99, 95% CI: 0.63, 3.35) brain volumes in children at ages 10 and 14 y (for example, total brain volume B: 4.01, 95% CI: 0.72, 7.3), and larger subcortical brain volume in children at age 10 y (B: 0.16, 95% CI: 0.04, 0.28). All associations remained significant after correction for multiple testing. When we additionally adjusted the models for child diet quality at age 8 y in the model 2, the effect estimates attenuated, but the associations of maternal diet quality with offspring brain volumes at age 10 y remained statistically significant.

**TABLE 2:** Associations of diet quality during pregnancy with child brain volumes at ages 10 and 14 y^1^.

	Model 1		Model 2	
	B	95% CI	B	95% CI
Total brain volume (cm^3^)				
At 10 y	4.54	(1.80, 7.28)^2^	4.16	(1.37, 6.96)^2^
At 14 y	4.01	(0.72, 7.30)^2^	3.47	(0.10, 6.85)
Cerebral white matter volume (cm^3^)				
At 10 y	1.83	(0.56, 3.10)^2^	1.71	(0.42, 3.00)^2^
At 14 y	1.69	(0.11, 3.27)^2^	1.51	(−0.11, 3.14)
Cerebral gray matter volume (cm^3^)				
At 10 y	1.99	(0.63, 3.35)^2^	1.75	(0.37, 3.14)^2^
At 14 y	1.93	(0.36, 3.50)^2^	1.65	(0.05, 3.26)
Subcortical volume (cm^3^)				
At 10 y	0.16	(0.04, 0.28)^2^	0.15	(0.02, 0.27)^2^
At 14 y	0.15	(0.00, 0.29)	0.11	(−0.04, 0.26)

Abbreviations: CI, confidence interval; FDR, false discovery rate.

1 The effect estimates represent the difference in cubic centimeters for children’s brain volumes per 1-unit higher score on the diet quality during pregnancy. Model 1 is the main model, adjusted for child sex and age when brain imaging was assessed, age of mother, maternal education, maternal national origin, and household income, smoking during pregnancy, maternal psychopathological symptoms during pregnancy, and mother’s energy intake. Model 2 was additionally adjusted for child diet quality at 8 y of age.

2 The associations that remained statistically significant after the Benjamini–Hochberg correction for multiple testing (8 tests) with an FDR ≤ 0.05.

**FIGURE 1:**
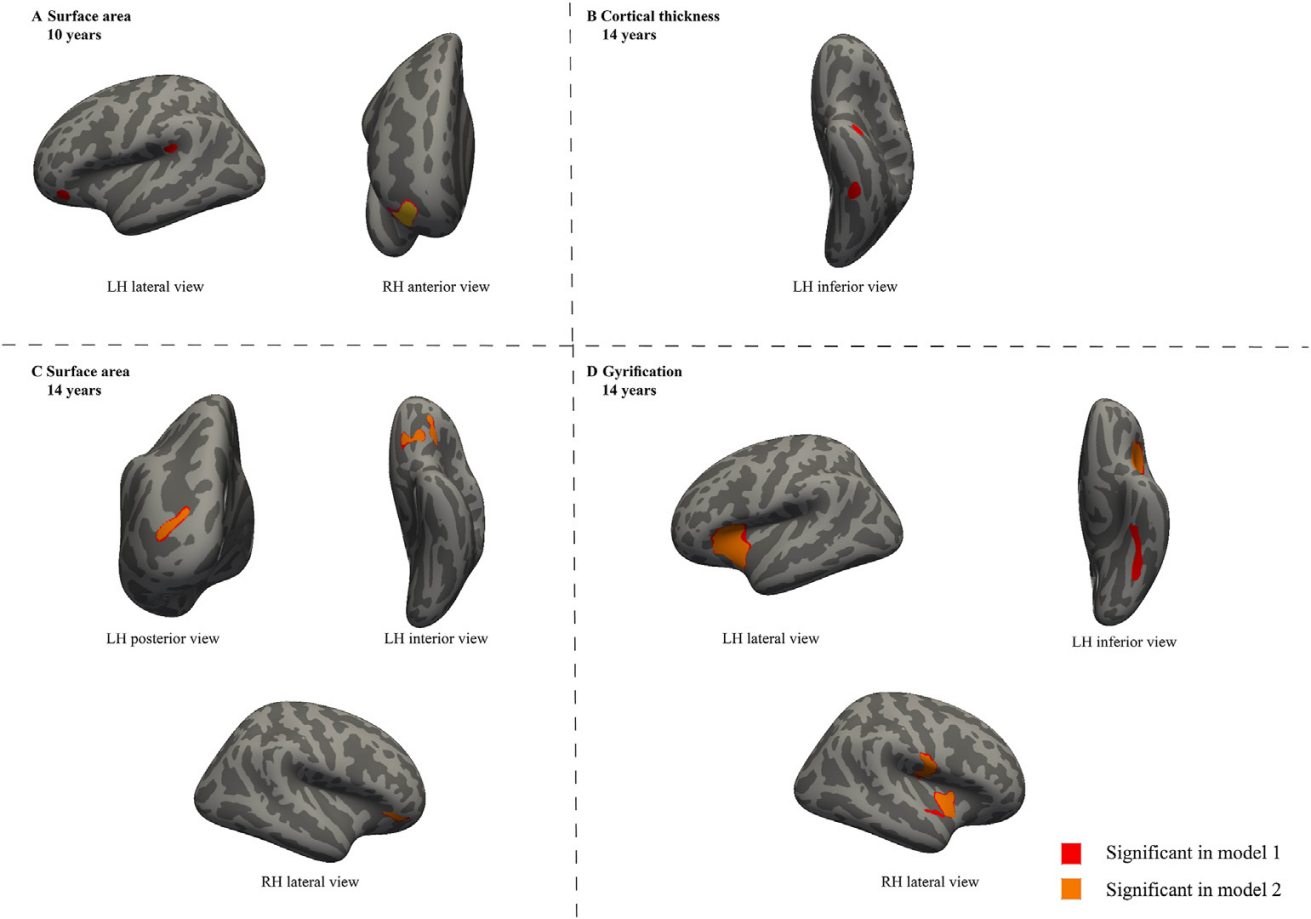
The associations of diet quality during pregnancy and surface-based brain morphology. Colored clusters represent (A) cortical thickness, (B) surface area, or (C) gyrification in regions of the brain that were positively associated with diet quality during pregnancy after the cluster-wise correction for multiple comparisons (*P* <0.001). Model 1 was adjusted for child sex and age when brain imaging was assessed, age of mother, maternal education, maternal ethnic background, and household income, smoking during pregnancy, maternal psychopathological symptoms during pregnancy, and mother’s energy intake. Model 2 was additionally adjusted for child diet quality at age 8 y. LH, left hemisphere; RH, right hemisphere.

Figure 1 shows that diet quality during pregnancy was also associated with widespread differences in cortical thickness, gyrification, and surface area in both hemispheres (model 1), which was seemingly independent of child diet quality at age 8 y (model 2), except for cortical thickness. Better diet quality during pregnancy was associated with a larger surface area in the lateral and inferior frontal regions in the left hemisphere, and rostral frontal region in the right hemisphere measured at age 10 y (Figure 1A). For surface-based morphometries measured at age 14 y, better diet quality during pregnancy was associated with smaller cortical thickness in the inferior region in the left hemisphere (Figure 1B), with larger surface area in the lateral orbitofrontal and occipital regions in the left hemisphere and the orbital part of inferior frontal region in the right hemisphere (Figure 1C), with greater gyrification in the anterior insula region in both hemispheres (Figure 1D). Specific information on the associated anatomical regions and their cluster-wise *P* values are presented in the Supplemental Table 3.

### Associations of diet quality during pregnancy with child cognitive performance and mediation by brain morphology

Table 3 shows the associations between diet quality during pregnancy and child cognitive performance (full-scale IQ and subtests) at age 14 y. After correcting for multiple testing, we found that better diet quality during pregnancy was associated with higher full-scale IQ (B: 0.65, 95% CI: 0.23, 1.08), which was mainly driven by higher subtest scores in vocabulary (B: 0.14, 95% CI: 0.05, 0.24) and matrix reasoning (B: 0.16, 95% CI: 0.07, 0.25). These associations remained after additional adjustment for child diet quality at age 8 y.

**TABLE 3:** Associations of diet quality during pregnancy with child IQ at 14 y (*n* = 2223)^1^.

	Model 1		Model 2	
	B	95% CI	B	95% CI
Full-scale IQ score	0.65	(0.23, 1.08)^2^	0.58	(0.15, 1.02)^2^
Vocabulary	0.14	(0.05, 0.24)^2^	0.12	(0.02, 0.21)^2^
Matrix reasoning	0.16	(0.07, 0.25)^2^	0.15	(0.06, 0.24)^2^
Digital span	0.07	(−0.02, 0.17)	0.07	(−0.03, 0.17)
Coding	0.03	(−0.08, 0.14)	0.03	(−0.08, 0.14)

Abbreviations: CI, confidence interval; FDR, false discovery rate; IQ, intelligence quotient.

1 The effect estimates represent the difference in IQ or subtest t-scores per 1-unit higher score on the diet quality during pregnancy. Model 1 is the main model, adjusted for child sex and age when brain imaging was assessed, age of mother, maternal education, maternal national origin, and household income, smoking during pregnancy, maternal psychopathological symptoms during pregnancy, and mother’s energy intake. Model 2 was additionally adjusted for child diet quality at 8 y of age.

2 The associations that remained statistically significant after the Benjamini–Hochberg correction for multiple testing (5 tests) with a FDR ≤ 0.05.

**FIGURE 2:**
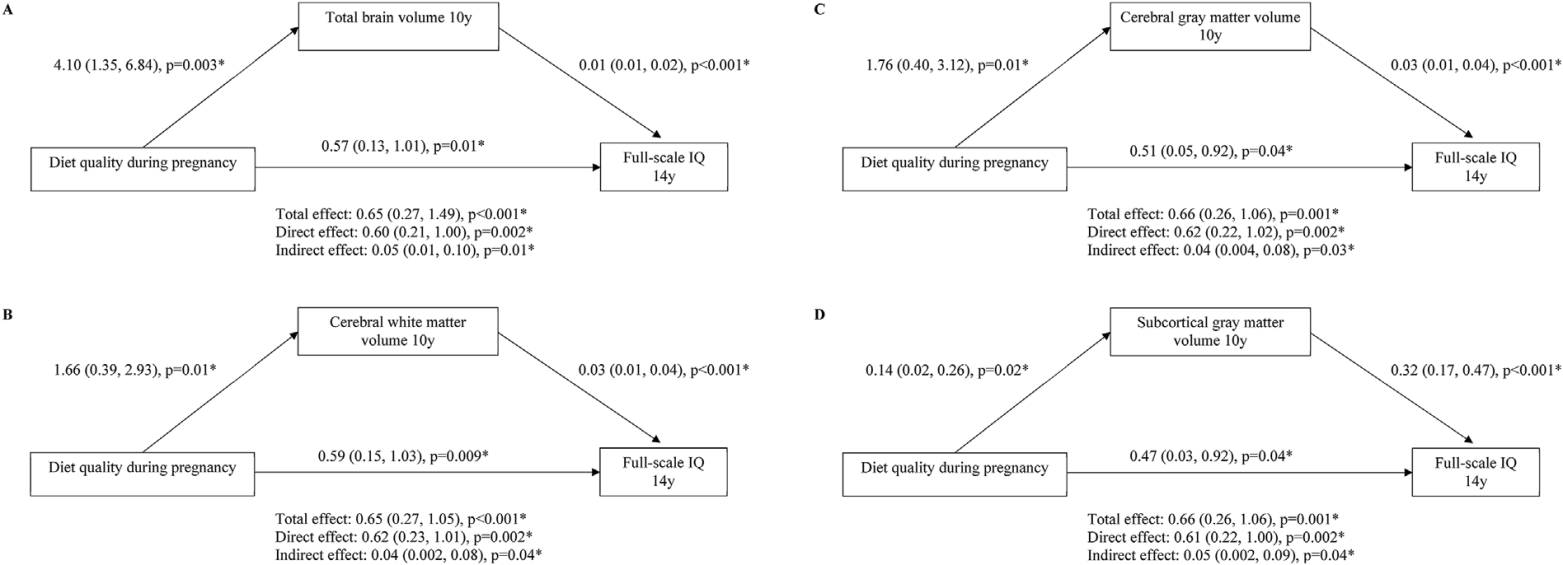
Global brain volumes mediating the association between diet quality during pregnancy and full-scale IQ at age 14 y. The association between diet quality during pregnancy and full-scale IQ at age 14 y was partially mediated by (A) total brain volume, (B) cerebral white matter volume, (C) cerebral gray matter volume, and (D) subcortical gray matter volume. Values represent the estimated coefficients (95% CIs) for each pathway, adjusted for child sex and age when brain imaging was assessed, age of mother, maternal education, maternal ethnic background, and household income, smoking during pregnancy, maternal psychopathological symptoms during pregnancy, and mother’s energy intake. ∗ denotes the estimate being statistically significant. CI, confidence interval; IQ, intelligence quotient.

We then examined whether brain volumetric measures mediated the associations of diet quality during pregnancy and child full-scale IQ. We observed that total brain, cerebral white matter, cerebral gray matter, and subcortical matter volumes all partially mediated the relationships between diet quality during pregnancy and child IQ at age 14 y (Figure 2). The proportions of the mediation effect ranged from 6.1% for the path through cerebral gray matter volume to 7.7% for the path through total brain volume.

### Sensitivity analysis

The non-response analysis showed that mothers who were included in our analyses tended to more often of have a Dutch national origin, and to have a higher socioeconomic status and higher diet quality score during pregnancy than those with missing data on FFQ and neuroimaging data at both ages (Supplemental Table 4). The analyses stratified by national origin of mothers showed larger effect estimates for the non-Dutch group (Supplemental Tables 5 and 6). Adjusting for breastfeeding duration slightly attenuated effect sizes of the associations, but the statistical significance remained the same (Supplemental Table 7). Results from linear mixed models with random intercepts to assess the longitudinal associations were similar (Supplemental Table 8). After adjusting for intracranial volume in the analyses of brain volumes, all associations attenuated and none remained statistically significant. This suggests that the observed differences in global brain volumes may be proportionally related to head size, indicating that brain volumetric changes in children aged 10 and 14 y may present global changes in relation to diet quality during pregnancy (Supplemental Table 9). When adding maternal gestational folate level in the models, we observed that the estimates were similar to the main results (Supplemental Table 10). Finally, the patterns of change in effect estimates from excluding each of the 15 food components one at a time and additionally adjusting for the excluded component in regression models indicate that the observed associations were not driven by any single food component (Supplemental Tables 11–13).

## Discussion

In a prospective population-based study, we observed a long-term association of diet quality during pregnancy, reflecting adherence to national dietary guidelines, on differences in global brain volumes and widespread differences in cortical thickness, surface area, and gyrification of children in pre- and early adolescence. Mothers’ diet quality during pregnancy was associated with total, cerebral white, and cerebral gray matter volume of their children at age 10 y and the associations persisted at age 14 y. These associations attenuated slightly after including child diet quality in mid-childhood. We observed that better diet quality during pregnancy was associated with higher full-scale IQ test scores, particularly in vocabulary and matrix reasoning subtests. Furthermore, we found that the association of diet quality during pregnancy on full-scale IQ in early adolescence was partially mediated by these brain volumetric markers in pre-adolescence. Notably, these associations were not driven by single food component, emphasizing the role of overall diet during pregnancy on child brain development in the setting of nourished population. Although the effect sizes were small, these findings potentially have substantial public health implications because prenatal nutrition is a modifiable factor with the potential for long-term impact on child brain development and cognitive outcomes.

This is the first epidemiological study to investigate the long-term associations between diet quality during pregnancy and children’s brain morphology. Our findings extend previous studies reporting that dietary patterns during pregnancy can impact child cognitive outcomes in mid-childhood [11,19,20] by showing that the association persists in early adolescence and by showing that this is partially mediated by brain morphological measures, suggesting that measurable neurobiological pathways underlie these associations. The associations between diet quality during pregnancy and child brain volumes are likely to be global over the long term, given that we observed consistent associations at ages 10 and 14 y. Although we only assessed diet quality in the first trimester, during which the neural tube forms and the foundation of the neuronal system are established, the observed associations could potentially reflect and extend to the entire period from preconception to birth. This can be supported by recent studies that have found a high level of stability in diet quality trajectories from preconception onwards [50,51]. Moreover, the associated brain clusters were distributed widely over the cerebrum. The significant brain regions are functionally linked with a wide range of cognitive functions. For example, the anterior insula cortex is suggested to mark salient information by referring to subjective feeling states and initiates cognitive processes for further processing the information [52]. The lateral orbitofrontal cortex is an important region involved in the emotion regulation and reward-processing: it receives and integrates input from sensory modalities and modulates decision-making behaviors [53]. Nevertheless, the degree to which functional connectivity corresponds to variations in brain morphology remains uncertain, despite theories suggesting a relationship between cortical morphology and enhanced connectivity [54]. Therefore, caution is needed regarding the translation of findings from functional connectivity studies with the observed association between diet quality during pregnancy and brain morphology.

We found that additional adjustment for child diet quality in mid-childhood slightly attenuated the effect estimates between diet quality during pregnancy and child's brain volumes and cognitive performance. This slight attenuation in effect sizes is consistent with our previous findings, where we demonstrated associations between diet quality in mid-childhood and brain morphological measures, as well as full-scale IQ, independent of diet quality during pregnancy [30]. Moreover, the results for brain regions found in this study remained when adjusting for child diet quality, and the statistically significant areas did not overlap with what we found in the previous study on child diet, suggesting a distinctive association of diet quality during pregnancy on brain morphology. Previous studies have rarely accounted for child diet quality when examining the association of maternal diet during pregnancy with downstream effects on child cognition. Our results provide a less biased estimate of the direct association of diet quality during pregnancy with child’s brain and cognitive development.

The etiology of the relationships between prenatal diet quality and neurodevelopment is not fully understood. Nonetheless, prior animal and human studies have proposed several mechanisms. An optimal prenatal diet quality may create a more favorable in utero nutritional profile, with many nutrients such as protein, iron, zinc, copper, selenium, vitamins A and B6, and folate being crucial for various neurodevelopmental processes [3,5]. In the Generation R Study, a higher diet quality score during pregnancy was positively correlated with these macro- and micronutrients [35]. Epigenetic modification presents a possible mediating mechanism underlying the effect of diet during pregnancy and neurodevelopment. Maternal nutrition during pregnancy may affect early epigenetic processes in the fetus, thus resulting in gene expression changes in the neurodevelopmental processes [55], which could lead to alterations in the brain structural organization. Diet-induced inflammation during pregnancy presents an alternative biological mechanism. Studies have shown that lower maternal diet quality in pregnancy may increase inflammation, as indicated by various elevated inflammatory markers concentration [56], potentially harming child neurodevelopment [57].

Strengths of our study are the prospective population-based study design, the inclusion of multiple covariates, and the large-scale and repeated pediatric neuroimaging data available. We assessed diet quality according to Dutch dietary guidelines, reflecting adherence to a healthy diet as determined by expert consensus. Furthermore, we included diet quality in mid-childhood when investigating the associations, providing insight into the independent impact of diet quality during pregnancy on child’s brain and cognitive outcomes.

Nevertheless, it is important to interpret our results within the context of several limitations. First, the use of FFQs to assess dietary intake is subject to measurement error. However, the FFQs used in this study were extensive, developed specifically for pregnant individuals, and validated against 24-h recalls and nutrient biomarkers among Dutch pregnant individuals [35], showing moderate to high validity for energy intake and intake of various nutrients. Moreover, the measurement error would likely have attenuated our effect estimates, as mothers with lower compliance to dietary recommendations tend to underreport their energy intake [58]. Second, the diet quality score used in this study was developed to measure adherence to Dutch dietary guidelines and was not validated against other diet quality indices. Although the dietary recommendations are generally similar to guidelines in other western countries, the score itself may not be directly applicable and may need modification if used in other populations. Nonetheless, we evaluated the diet quality score with nutrient intake and found associations of the score with nutrients in the expected direction, confirming desirable internal validity of the diet quality score. Third, although we adjusted for several covariates including socioeconomic status, maternal psychopathology and lifestyle factors, we cannot dismiss the possibility of genetic and unmeasured environmental confounding (for example, stimuli in home environment) because of the observational nature of the study. Fourth, diet quality typically decreases and shows greater variability during adolescence compared with childhood. Therefore, including diet quality of adolescents, the data we lack, could further attenuate the associations between prenatal diet quality and child brain morphology and IQ at 14 y of age. Lastly, the non-response analyses suggested a potential selection bias, which may limit the establishment of generalizability.

In conclusion, our findings suggest potential long-term and global effects of diet quality during pregnancy on brain development and cognitive performance through early adolescence. Future studies are warranted to replicate our findings in different populations and delve deeper into regional differences in brain morphological measures, while also considering the effect of child diet quality to distinguish the direct effect of prenatal diet quality from later dietary exposures. Finally, it is important to learn whether additional consequences of these neurodevelopmental effects will occur with further aging, such as an increase in psychiatric disorders or cognitive problems during adolescence and adulthood.

## Author contributions

The authors’ responsibilities were as follows – YM, TV: designed research; TW, TV: provided essential material (databases); YM: performed main statistical analysis; HS: performed additional statistical analysis; PWJ, TW, TV: provided consultation of results interpretation; YM: wrote the original draft; YM, PWJ, HS, TW, TV: reviewed and edited the article; YM, TV: had primary responsibility for final content; and all authors: read and approved the final manuscript.

## Funding

The general design of the Generation R Study is made possible by financial support from the Erasmus MC, University Medical Center, Rotterdam, Erasmus University Rotterdam, The Netherlands Organization for Health Research and Development (ZonMw), Netherlands Organization for Scientific Research (NWO), Ministry of Health, Welfare and Sport, and Ministry of Youth and Families. YM is supported by China Scholarship Council (CSC) PhD Fellowship (201806240125) for her PhD study in Erasmus MC, University Medical Center Rotterdam. PJ is supported by a grant from ZonMw (Mental Health Care Research Program—Fellowship 636320005). HS is supported by CSC PhD Fellowship (202206240031) for her PhD study in Erasmus MC, University Medical Center Rotterdam. The neuroimaging data collection and image processing is supported by ZonMw TOP Grant 91211021 to TW, who also is receiving support from the Intramural Research Program of the National Institute of Mental Health. Supercomputing resources were supported by the NWO Physical Sciences Division (Exacte Wetenschappen) and SURFsara (Cartesius compute cluster, www.surfsara.nl). The funders had no role in the study design, data collection, management, analysis and interpretation of data, and preparation or writing the manuscript.

## Data availability

Data described in the manuscript cannot be made publicly available because of confidentiality, but data can be requested and shared with a formal data-sharing agreement. Requests for data, code book, and analytic code can be directed to datamangementgenr@erasmusmc.nl.

## Conflict of interest

The authors report no conflicts of interest.
