# Supplementary material for: Diet quality during pregnancy, adolescent brain morphology, and cognitive performance in a population-based cohort
Source: Am J Clin Nutr. 2024 Oct 8;120(5):1125–33. doi: 10.1016/j.ajcnut.2024.08.018 (PMC13169018; doi:10.1016/j.ajcnut.2024.08.018)
Supplement: multimedia component 1 [file mmc1.docx]

# Online Supplementary Material

**Diet quality during pregnancy, and child brain morphology and cognitive performance in a population-based cohort**

Yuchan Mou, et al.

**Content**

Supplemental Table 1. Correlations between the diet quality score and intake of nutrients

Supplemental Table 2. Numbers and percentages of missing values of covariates

Supplemental Table 3. Brain regions that are associated with diet quality during pregnancy and the corresponding p values

Supplemental Table 4. Non-response analysis

Supplemental Table 5. Association of diet quality during pregnancy with child global brain volumes at ages 10 and 14 years in mothers with Dutch national origin

Supplemental Table 6. Association of diet quality during pregnancy with child global brain volumes at ages 10 and 14 years in mothers with non-Dutch national origin

Supplemental Table 7. Associations of diet quality during pregnancy with child global brain volumes and IQ additionally adjusted for breastfeeding duration

Supplemental Table 8. Associations of diet quality during pregnancy with child global brain volumes using linear mixed model

Supplemental Table 9. Association of diet quality during pregnancy with child global brain volumes at ages 10 and 14 years adjusted for intracranial volumes

Supplemental Table 10. Associations of diet quality during pregnancy with child global brain volumes and IQ additionally adjusted for maternal gestational folate concentration

Supplemental Table 11. Associations of diet quality during pregnancy with child brain volumes at ages 10 years after excluding each of 15 components one at a time, and additionally adjusting for the excluded one

Supplemental Table 12. Associations of diet quality during pregnancy with child brain volumes at ages 14 years after excluding each of 15 components one at a time, and additionally adjusting for the excluded one

Supplemental Table 13. Associations of diet quality during pregnancy with child IQ at 14 years after excluding each of 15 components one at a time, and additionally adjusting for the excluded one

Supplemental Figure 1. Study population flowchart

Supplemental Table 1. Correlations between the diet quality score and intake of nutrients

|  | | Pearson correlation (*r*)  Unadjusted | Pearson correlation (*r*)  Adjusted for energy intake |
| --- | --- | --- | --- |
| Macronutrients: | |  |  |
| Fat (g) | | 0.27 | 0.01 |
|  | Saturated fat (g) | 0.17 | -0.14 |
|  | Monounsaturated Fat (g) | 0.28 | 0.06 |
|  | Polyunsaturated Fat (g) | 0.29 | 0.12 |
| Protein (g) | | 0.40 | 0.29 |
|  | Animal protein (g) | 0.21 | 0.02 |
|  | Plant protein (g) | 0.54 | 0.49 |
| Carbohydrates (g) | | 0.24 | -0.09 |
|  | Monosaccharides and disaccharides (g) | 0.14 | -0.14 |
|  | Total Polysaccharides (g) | 0.30 | 0.09 |
|  | Dietary fiber (g) | 0.64 | 0.59 |
| Micronutrients: | |  |  |
| Vitamin B2 (mg) | | 0.33 | 0.18 |
| Vitamin B6 (mg) | | 0.37 | 0.22 |
| Vitamin C (mg) | | 0.24 | 0.12 |
| Calcium (mg) | | 0.44 | 0.31 |
| Iron (mg) | | 0.47 | 0.38 |
| Sodium (mg) | | 0.07 | -0.11 |
| Potassium (mg) | | 0.43 | 0.31 |
| Magnesium (mg) | | 0.58 | 0.57 |
| Phosphorus (mg) | | 0.51 | 0.44 |
| Zinc (mg) | | 0.44 | 0.33 |

Supplemental Table 2. Numbers and percentages of missing values of covariates

|  | Sample with data at 10 years  n = 2223 |  | Sample with data at 14 years  n= 1582 |
| --- | --- | --- | --- |
|  | Missing values n (%) | | |
| *Maternal characteristics* |  |  |  |
| Age at enrollment | - |  | - |
| Educational level | 189 (8.4) |  | 128 (8.0) |
| Household income per month | 244 (10.9) |  | 135 (8.5) |
| National origin | 12 (0.5) |  | 13 (0.8) |
| Smoking during pregnancy | 196 (8.7) |  | 135 (8.5) |
| Psychological symptoms | 266 (11.8) |  | 208 (13.1) |
|  |  |  |  |
| *Child characteristics* |  |  |  |
| Age at the neuroimaging assessment | - |  | - |
| Sex | - |  | - |
| Diet quality | 516 (22.9) |  | 404 (25.4) |

Supplemental Table 3. Brain regions that are associated with diet quality during pregnancy and the corresponding *p* values^1^

| Cortical surface measures | Hemisphere | Anatomical Region | | Area Size (mm^2^) | | Coordinates | | | | | | Mean coefficient | | Cluster-wise *p* value |
| --- | --- | --- | --- | --- | --- | --- | --- | --- | --- | --- | --- | --- | --- | --- |
|  |  |  | |  | | x | | y | | z | |  | |  |
| 10 years |  |  | |  | |  | |  | |  | |  | |  |
| Surface area | LH | Pars orbitalis | | 162.64 | | -42.4 | | 35.3 | | -14.2 | | 0.009 | | 0.016 |
|  |  | Supramarginal | | 153.62 | | -43.0 | | -36.0 | | 23.3 | | 0.007 | | 0.021 |
|  | RH | Rostral middle frontal | | 356.05 | | 26.4 | | 57.9 | | -9.2 | | 0.015 | | <0.001 |
| 14 years | | | | | | | | | | | | | | |
| Surface area | LH | Lateral orbitofrontal | 220.94 | | -27.2 | | 26.5 | | -15.8 | | 0.011 | | 0.003 | |
|  |  | Lateral occipital | 204.69 | | -28.2 | | -88.7 | | 7.2 | | 0.014 | | 0.004 | |
|  |  | Lateral orbitofrontal | 202.73 | | -41.9 | | 26.5 | | -14.1 | | 0.010 | | 0.005 | |
|  | RH | Pars orbitalis | 215.55 | | 47.4 | | 32.3 | | -12.3 | | 0.014 | | 0.003 | |
| Gyrification | LH | Insula | 1411.14 | | -31.2 | | 19.7 | | -3.8 | | 0.026 | | <0.001 | |
|  |  | Fusiform | 483.24 | | -37.0 | | -29.9 | | -25.2 | | 0.008 | | 0.006 | |
|  | RH | Insula | 542.50 | | 36.5 | | -3.5 | | -2.9 | | 0.025 | | 0.003 | |
|  |  | Postcentral | 507.34 | | 61.7 | | -6.1 | | 10.4 | | 0.023 | | 0.004 | |
| Cortical thickness | LH | Inferior temporal | 164.37 | | -52.4 | | -37.4 | | -27.0 | | -0.021 | | 0.024 | |

^1^Abbreviations: RH, right hemisphere; LH, left hemisphere. The models were adjusted for child sex and age when brain imaging was assessed, maternal education, household income, child national origin, child energy intake, child BMI measured at the age of 10 years, maternal diet quality during pregnancy, smoking during pregnancy, alcohol use during pregnancy, folic acid use, and maternal psychopathological symptoms during pregnancy.

Supplemental Table 4. Non-response analysis^1^

|  | Sample with data at 10 years | |  | Sample with data at 14 years | |
| --- | --- | --- | --- | --- | --- |
|  | Respondents  (n = 2223) | Non-Respondents  (n = 4262) |  | Respondents  (n = 1582) | Non-Respondents  (n = 4903) |
| Maternal characteristics |  |  |  |  |  |
| Age at enrollment | 31.2 (4.6) | 29.6 (5.2) |  | 31.2 (4.7) | 29.8 (5.1) |
| Educational level (High), N (%) | 1352 (66.4) | 1917 (49.4) |  | 941 (64.7) | 2328 (52.2) |
| Household income per month, N (%) |  |  |  |  |  |
| < 1200 € | 207 (10.5) | 656 (19.6) |  | 152 (10.8) | 711 (18.1) |
| 1200 – 2200 € | 416 (21.0) | 870 (26.0) |  | 325 (23.2) | 961 (24.5) |
| > 2200 € | 1357 (68.5) | 1817 (54.4) |  | 926 (66.0) | 2248 (57.3) |
| National origin, N (%) |  |  |  |  |  |
| Dutch | 1416 (64.0) | 2194 (53.0) |  | 969 (61.8) | 2641 (55.2) |
| Non-Dutch | 796 (36.0) | 1944 (47.0) |  | 600 (38.2) | 2140 (44.8) |
| Smoking during pregnancy, N (%) |  |  |  |  |  |
| Never | 1602 (78.9) | 2762 (70.8) |  | 1125 (77.6) | 3239 (72.3) |
| Until pregnancy was known | 183 (9.0) | 345 (8.8) |  | 116 (8.0) | 412 (9.2) |
| Continued | 245 (12.1) | 795 (20.4) |  | 209 (14.4) | 831 (18.5) |
| Diet quality during pregnancy | 7.8 (1.6) | 7.4 (1.7) |  | 7.7 (1.6) | 7.5 (1.7) |
|  |  |  |  |  |  |
| Child characteristics |  |  |  |  |  |
| Sex (Girls), N (%) | 1122 (50.5) | 2050 (48.9) |  | 850 (53.8) | 2322 (48.1) |
| Diet quality scores at age eight years | 4.6 (1.2) | 4.5 (1.2) |  | 4.5 (1.2) | 4.5 (1.2) |

^1^Values are mean (SD) for continuous variables with a normal distribution, medians (IQR) for continuous variables with a skewed distribution, or valid numbers n (%) for categorical variables. Continuous variables are presented as mean (SD) or as median (IQR) and compared; categorical variables were presented as number (%). Statistics were based on the non-imputed data.

Supplemental Table 5. Association of diet quality during pregnancy with child global brain volumes at ages 10 and 14 years in mothers with a Dutch national origin only^1^

|  | Model 1 | |  | Model 2 | |
| --- | --- | --- | --- | --- | --- |
|  | *B* | 95% CI |  | *B* | 95% CI |
|  |  |  |  |  |  |
| Total brain volume (cm^3^) |  |  |  |  |  |
| at 10 years | 2.90 | (-0.70, 6.50) |  | 1.94 | (-1.80, 5.68) |
| at 14 years | 4.11 | (-0.34, 8.55) |  | 2.52 | (-2.13, 7.16) |
| Cerebral white matter volume (cm^3^) |  |  |  |  |  |
| at 10 years | 1.15 | (-0.52, 2.83) |  | 0.76 | (-0.98, 2.50) |
| at 14 years | 1.91 | (-0.24, 4.06) |  | 1.29 | (-0.96, 3.54) |
| Cerebral gray matter volume (cm^3^) |  |  |  |  |  |
| at 10 years | 1.17 | (-0.61, 2.96) |  | 0.68 | (-1.18, 2.53) |
| at 14 years | 1.93 | (-0.18, 4.05) |  | 1.18 | (-1.03, 3.40) |
| Subcortical volume (cm^3^) |  |  |  |  |  |
| at 10 years | 0.12 | (-0.04, 0.27) |  | 0.08 | (-0.08, 0.24) |
| at 14 years | 0.19 | (-0.01, 0.39) |  | 0.10 | (-0.11, 0.31) |

^1^The effect estimates represent the difference in cubic centimeters for brain volumes per 1-unit higher score on the diet quality during pregnancy. Model 1 adjusted for child sex and age when brain imaging was assessed, age of mother, maternal education, maternal national origin, and household income, smoking during pregnancy, maternal psychopathological symptoms during pregnancy, and mother’s energy intake. In addition to model 1, model 2 was additionally adjusted for child diet quality at eight years of age.

Supplemental Table 6. Association of diet quality during pregnancy with child global brain volumes at ages 10 and 14 years in mothers with a non-Dutch national origin^1^

|  | Model 1 | |  | Model 2 | |
| --- | --- | --- | --- | --- | --- |
|  | *B* | 95% CI |  | *B* | 95% CI |
|  |  |  |  |  |  |
| Total brain volume (cm^3^) |  |  |  |  |  |
| at 10 years | 6.06 | (1.69, 10.43) |  | 5.71 | (1.21, 10.20) |
| at 14 years | 3.58 | (-1.57, 8.73) |  | 3.36 | (-2.02, 8.74) |
| Cerebral white matter volume (cm^3^) |  |  |  |  |  |
| at 10 years | 2.59 | (0.60, 4.59) |  | 2.55 | (0.50, 4.60) |
| at 14 years | 1.40 | (-1.04, 3.84) |  | 1.37 | (-1.17, 3.9) |
| Cerebral gray matter volume (cm^3^) |  |  |  |  |  |
| at 10 years | 2.58 | (0.41, 4.74) |  | 2.31 | (0.08, 4.53) |
| at 14 years | 1.56 | (-0.94, 4.06) |  | 1.42 | (-1.19, 4.02) |
| Subcortical volume (cm^3^) |  |  |  |  |  |
| at 10 years | 0.18 | (-0.02, 0.38) |  | 0.17 | (-0.03, 0.38) |
| at 14 years | 0.06 | (-0.18, 0.29) |  | 0.04 | (-0.21, 0.29) |

^1^The effect estimates of diet quality score represent the difference in cubic centimeters for brain volumes per 1-unit higher score on the diet quality during pregnancy. Model 1 adjusted for child sex and age when brain imaging was assessed, age of mother, maternal education, maternal national origin, and household income, smoking during pregnancy, maternal psychopathological symptoms during pregnancy, and mother’s energy intake. In addition to model 1, model 2 was additionally adjusted for child diet quality at eight years of age.

Supplemental Table 7. Associations of diet quality during pregnancy with child global brain volumes and IQ additionally adjusted for breastfeeding duration^1^

|  | Model 1 | |  | Model 2 | |
| --- | --- | --- | --- | --- | --- |
|  | *B* | 95% CI |  | *B* | 95% CI |
| ***Brain volumes*** |  |  |  |  |  |
| Total brain volume (cm^3^) |  |  |  |  |  |
| at 10 years | 4.42 | (1.68, 7.15) |  | 4.06 | (1.26, 6.86) |
| at 14 years | 4.01 | (0.72, 7.30) |  | 3.48 | (0.09, 6.88) |
| Cerebral white matter volume (cm^3^) |  |  |  |  |  |
| at 10 years | 1.79 | (0.52, 3.06) |  | 1.68 | (0.38, 2.97) |
| at 14 years | 1.69 | (0.11, 3.27) |  | 1.54 | (-0.10, 3.17) |
| Cerebral gray matter volume (cm^3^) |  |  |  |  |  |
| at 10 years | 1.90 | (0.54, 3.26) |  | 1.68 | (0.30, 3.07) |
| at 14 years | 1.93 | (0.36, 3.50) |  | 1.62 | (0.00, 3.24) |
| Subcortical volume (cm3) |  |  |  |  |  |
| at 10 years | 0.16 | (0.04, 0.28) |  | 0.14 | (0.02, 0.27) |
| at 14 years | 0.15 | (0.00, 0.29) |  | 0.11 | (-0.04, 0.26) |
| ***IQ*** |  |  |  |  |  |
| Full-scale IQ score | 0.63 | (0.21, 1.05) |  | 0.56 | (0.13, 1.00) |
| Vocabulary | 0.14 | (0.05, 0.23) |  | 0.11 | (0.02, 0.21) |
| Matrix reasoning | 0.15 | (0.07, 0.24) |  | 0.14 | (0.05, 0.23) |
| Digital span | 0.07 | (-0.03, 0.17) |  | 0.07 | (-0.03, 0.17) |
| Coding | 0.03 | (-0.08, 0.14) |  | 0.03 | (-0.08, 0.14) |

^1^Abbreviations: CI, confidence interval; IQ, intelligence quotient. The effect estimates of diet quality score represent the difference in cubic centimeters for brain volumes/the difference in IQ or subtest t scores per 1-unit higher score on the diet quality during pregnancy. Model 1 adjusted for child sex and age when brain imaging was assessed, age of mother, maternal education, maternal national origin, and household income, smoking during pregnancy, maternal psychopathological symptoms during pregnancy, mother’s energy intake, and breastfeeding duration. In addition to model 1, model 2 was additionally adjusted for child diet quality at eight years of age.

Supplemental Table 8. Associations of diet quality during pregnancy with child global brain volumes using linear mixed model^1^

|  | Model 1 | |  | Model 2 | |
| --- | --- | --- | --- | --- | --- |
|  | *B* | 95% CI |  | *B* | 95% CI |
| Total brain volume (cm^3^) | 5.93 | (1.68, 10.17) |  | 5.49 | (1.16, 9.83) |
| Cerebral white matter volume (cm^3^) | 2.83 | (0.83, 4.83) |  | 2.71 | (0.66, 4.75) |
| Cerebral gray matter volume (cm^3^) | 2.56 | (0.52, 4.61) |  | 2.23 | (0.14, 4.31) |
| Subcortical volume (cm3) | 0.17 | (-0.02, 0.36) |  | 0.15 | (-0.05, 0.34) |

^1^The effect estimates of diet quality score represent the difference in cubic centimeters for brain volumes per 1-unit higher score on the diet quality during pregnancy. Model 1 adjusted for child sex and age when brain imaging was assessed, age of mother, maternal education, maternal national origin, and household income, smoking during pregnancy, maternal psychopathological symptoms during pregnancy, and mother’s energy intake. In addition to model 1, model 2 was additionally adjusted for child diet quality at eight years of age.

Supplemental Table 9. Association of diet quality during pregnancy with child global brain volumes at ages 10 and 14 years adjusted for intracranial volumes^1^

|  | Model 1 | |  | Model 2 | |
| --- | --- | --- | --- | --- | --- |
|  | *B* | 95% CI |  | *B* | 95% CI |
| Total brain volume (cm^3^) |  |  |  |  |  |
| at 10 years | 0.31 | (-0.87, 1.49) |  | 0.44 | (-0.76, 1.64) |
| at 14 years | -0.15 | (-1.68, 1.38) |  | -0.26 | (-1.83, 1.30) |
| Cerebral white matter volume (cm^3^) |  |  |  |  |  |
| at 10 years | 0.00 | (-0.69, 0.68) |  | 0.10 | (-0.60, 0.79) |
| at 14 years | -0.16 | (-1.07, 0.75) |  | -0.15 | (-1.08, 0.78) |
| Cerebral gray matter volume (cm^3^) |  |  |  |  |  |
| at 10 years | 0.06 | (-0.7, 0.82) |  | 0.06 | (-0.71, 0.83) |
| at 14 years | 0.10 | (-0.81, 1.02) |  | 0.02 | (-0.92, 0.95) |
| Subcortical volume (cm^3^) |  |  |  |  |  |
| at 10 years | 0.01 | (-0.08, 0.09) |  | 0.01 | (-0.07, 0.09) |
| at 14 years | -0.01 | (-0.11, 0.09) |  | -0.03 | (-0.13, 0.08) |

^1^The effect estimates of diet quality score represent the difference in cubic centimeters for brain volumes per 1-unit higher score on the diet quality during pregnancy. Model 1 adjusted for child sex and age when brain imaging was assessed, age of mother, maternal education, maternal national origin, and household income, smoking during pregnancy, maternal psychopathological symptoms during pregnancy, mother’s energy intake, and intracranial volumes. In addition to model 1, model 2 was additionally adjusted for child diet quality at eight years of age.

Supplemental Table 10. Associations of diet quality during pregnancy with child global brain volumes and IQ additionally adjusted for maternal gestational folate concentration^1^

|  | Model 1 | |  | Model 2 | |
| --- | --- | --- | --- | --- | --- |
|  | *B* | 95% CI |  | *B* | 95% CI |
| ***Brain volumes*** |  |  |  |  |  |
| Total brain volume (cm^3^) |  |  |  |  |  |
| at 10 years | 4.95 | (2.10, 7.80) |  | 4.66 | (1.75, 7.58) |
| at 14 years | 5.21 | (1.66, 8.75) |  | 4.71 | (1.05, 8.36) |
| Cerebral white matter volume (cm^3^) |  |  |  |  |  |
| at 10 years | 1.84 | (0.52, 3.16) |  | 1.74 | (0.40, 3.08) |
| at 14 years | 2.00 | (0.32, 3.69) |  | 1.83 | (0.08, 3.58) |
| Cerebral gray matter volume (cm^3^) |  |  |  |  |  |
| at 10 years | 2.31 | (0.90, 3.73) |  | 2.14 | (0.69, 3.59) |
| at 14 years | 2.58 | (0.88, 4.27) |  | 2.33 | (0.58, 4.07) |
| Subcortical volume (cm3) |  |  |  |  |  |
| at 10 years | 0.16 | (0.04, 0.29) |  | 0.15 | (0.03, 0.28) |
| at 14 years | 0.18 | (0.02, 0.35) |  | 0.15 | (-0.01, 0.32) |
| ***IQ*** |  |  |  |  |  |
| Full-scale IQ score | 0.60 | (0.13, 1.06) |  | 0.53 | (0.06, 1.01) |
| Vocabulary | 0.15 | (0.05, 0.25) |  | 0.12 | (0.02, 0.23) |
| Matrix reasoning | 0.15 | (0.06, 0.24) |  | 0.14 | (0.05, 0.23) |
| Digital span | 0.06 | (-0.04, 0.15) |  | 0.05 | (-0.05, 0.15) |
| Coding | 0.03 | (-0.09, 0.14) |  | 0.03 | (-0.09, 0.14) |

^1^Abbreviations: CI, confidence interval; IQ, intelligence quotient. The effect estimates of diet quality score represent the difference in cubic centimeters for brain volumes/the difference in IQ or subtest t scores per 1-unit higher score on the diet quality during pregnancy. Model 1 adjusted for child sex and age when brain imaging was assessed, age of mother, maternal education, maternal national origin, and household income, smoking during pregnancy, maternal psychopathological symptoms during pregnancy, mother’s energy intake, breastfeeding duration, and maternal gestational folate concentration. In addition to model 1, model 2 was additionally adjusted for child diet quality at eight years of age.

Supplemental Table 11. Associations of diet quality during pregnancy with child brain volumes at ages 10 years after excluding each of 15 components one at a time, and additionally adjusting for the excluded one^1^

| Diet quality score with 14 components instead of 15 components | Total brain volume (cm^3^) | Cerebral white matter volume (cm^3^) | Cerebral gray matter volume (cm^3^) | Subcortical volume (cm^3^) |
| --- | --- | --- | --- | --- |
| Excluding vegetables | 3.49 (0.56, 6.42) | 1.45 (0.09, 2.81) | 1.48 (0.03, 2.94) | 0.11 (-0.02, 0.24) |
| Excluding fruits | 3.77 (0.86, 6.68) | 1.42 (0.07, 2.77) | 1.63 (0.19, 3.08) | 0.12 (0.00, 0.25) |
| Excluding whole grains | 4.10 (1.08, 7.12) | 1.78 (0.39, 3.18) | 1.67 (0.18, 3.17) | 0.13 (0.00, 0.26) |
| Excluding legumes | 5.25 (2.36, 8.13) | 2.28 (0.94, 3.61) | 2.27 (0.84, 3.70) | 0.17 (0.04, 0.30) |
| Excluding nuts | 4.62 (1.74, 7.49) | 1.98 (0.65, 3.31) | 1.93 (0.50, 3.36) | 0.17 (0.04, 0.29) |
| Excluding dairy | 4.67 (1.87, 7.47) | 1.95 (0.66, 3.25) | 1.97 (0.58, 3.35) | 0.17 (0.05, 0.29) |
| Excluding fish | 3.86 (0.95, 6.77) | 1.40 (0.06, 2.75) | 1.82 (0.38, 3.26) | 0.14 (0.01, 0.27) |
| Excluding tea | 4.64 (1.75, 7.53) | 1.77 (0.44, 3.11) | 2.06 (0.63, 3.49) | 0.19 (0.06, 0.31) |
| Excluding grain quality | 4.56 (1.61, 7.51) | 1.88 (0.51, 3.25) | 1.95 (0.49, 3.41) | 0.17 (0.04, 0.30) |
| Excluding soft fats and oils | 5.21 (2.37, 8.06) | 2.21 (0.89, 3.52) | 2.23 (0.82, 3.64) | 0.18 (0.06, 0.31) |
| Excluding red meat | 4.84 (1.97, 7.71) | 1.87 (0.54, 3.20) | 2.23 (0.81, 3.66) | 0.17 (0.04, 0.30) |
| Excluding sugar-containing beverages | 4.90 (2.06, 7.74) | 2.01 (0.69, 3.32) | 2.10 (0.69, 3.51) | 0.16 (0.04, 0.29) |
| Excluding alcohol | 5.12 (2.24, 8.00) | 1.95 (0.61, 3.28) | 2.34 (0.92, 3.77) | 0.19 (0.06, 0.32) |
| Excluding salt | 4.41 (1.66, 7.15) | 1.77 (0.50, 3.04) | 1.93 (0.56, 3.29) | 0.15 (0.03, 0.28) |
| Excluding folic acid | 3.74 (0.83, 6.65) | 1.49 (0.14, 2.84) | 1.68 (0.24, 3.13) | 0.12 (-0.01, 0.25) |

^1^Effect estimates are regression coefficients (β) for child brain volumes at 10 years with their 95%-confidence intervals (95%CIs), per 1-unit higher score on the diet quality during pregnancy by excluding one of 15 foods at a time and additionally adjusting for the excluded food group. Model adjusted for child sex and age when brain imaging was assessed, age of mother, maternal education, maternal ethnic background, and household income, smoking during pregnancy, maternal psychopathological symptoms during pregnancy, and mother’s energy intake, based on pooled results of imputed data.

Supplemental Table 12. Associations of diet quality during pregnancy with child brain volumes at ages 14 years after excluding each of 15 components one at a time, and additionally adjusting for the excluded one^1^

| Diet quality score with 14 components instead of 15 components | Total brain volume (cm^3^) | Cerebral white matter volume (cm^3^) | Cerebral gray matter volume (cm^3^) | Subcortical volume (cm^3^) |
| --- | --- | --- | --- | --- |
| Excluding vegetables | 3.09 (-0.42, 6.61) | 1.26 (-0.43, 2.95) | 1.48 (-0.2, 3.16) | 0.09 (-0.06, 0.25) |
| Excluding fruits | 3.12 (-0.39, 6.63) | 1.26 (-0.43, 2.95) | 1.63 (-0.05, 3.30) | 0.11 (-0.04, 0.27) |
| Excluding whole grains | 3.82 (0.23, 7.41) | 1.81 (0.08, 3.54) | 1.56 (-0.16, 3.27) | 0.14 (-0.03, 0.30) |
| Excluding legumes | 4.42 (0.98, 7.87) | 1.97 (0.31, 3.62) | 2.13 (0.48, 3.77) | 0.15 (-0.01, 0.30) |
| Excluding nuts | 3.56 (0.08, 7.03) | 1.73 (0.06, 3.41) | 1.46 (-0.19, 3.12) | 0.14 (-0.02, 0.29) |
| Excluding dairy | 3.43 (0.08, 6.78) | 1.47 (-0.14, 3.09) | 1.63 (0.04, 3.23) | 0.13 (-0.02, 0.28) |
| Excluding fish | 4.08 (0.58, 7.57) | 1.62 (-0.06, 3.3) | 2.08 (0.41, 3.75) | 0.16 (0.00, 0.32) |
| Excluding tea | 4.02 (0.54, 7.50) | 1.58 (-0.09, 3.26) | 1.99 (0.33, 3.65) | 0.17 (0.01, 0.32) |
| Excluding grain quality | 3.84 (0.31, 7.37) | 1.73 (0.03, 3.43) | 1.64 (-0.04, 3.32) | 0.17 (0.01, 0.33) |
| Excluding soft fats and oils | 4.95 (1.54, 8.35) | 2.23 (0.59, 3.87) | 2.25 (0.63, 3.88) | 0.17 (0.02, 0.33) |
| Excluding red meat | 4.52 (1.06, 7.99) | 1.88 (0.21, 3.55) | 2.24 (0.58, 3.89) | 0.15 (0.00, 0.31) |
| Excluding sugar-containing beverages | 4.17 (0.74, 7.60) | 1.74 (0.09, 3.39) | 1.98 (0.35, 3.62) | 0.13 (-0.03, 0.28) |
| Excluding alcohol | 4.43 (0.96, 7.90) | 1.6 (-0.07, 3.27) | 2.39 (0.74, 4.04) | 0.17 (0.02, 0.33) |
| Excluding salt | 3.99 (0.70, 7.28) | 1.67 (0.09, 3.25) | 1.93 (0.36, 3.50) | 0.14 (0.00, 0.29) |
| Excluding folic acid | 4.04 (0.53, 7.55) | 1.74 (0.05, 3.43) | 1.91 (0.24, 3.58) | 0.14 (-0.02, 0.30) |

^1^Effect estimates are regression coefficients (β) for child brain volumes at 14 years with their 95%-confidence intervals (95%CIs), per 1-unit higher score on the diet quality during pregnancy by excluding one of 15 foods at a time and additionally adjusting for the excluded food group. Model adjusted for child sex and age when brain imaging was assessed, age of mother, maternal education, maternal ethnic background, and household income, smoking during pregnancy, maternal psychopathological symptoms during pregnancy, and mother’s energy intake, based on pooled results of imputed data.

Supplemental Table 13. Associations of diet quality during pregnancy with child IQ at 14 years after excluding each of 15 components one at a time, and additionally adjusting for the excluded one^1^

| Diet quality score with 14 components instead of 15 components | Full scale IQ score | Vocabulary | Matrix reasoning | Digital span | Coding |
| --- | --- | --- | --- | --- | --- |
| Excluding vegetables | 0.50 (0.00, 1.00) | 0.07 (-0.04, 0.17) | 0.13 (0.03, 0.22) | 0.12 (0.02, 0.23) | -0.01 (-0.13, 0.11) |
| Excluding fruits | 0.44 (-0.05, 0.94) | 0.14 (0.03, 0.25) | 0.13 (0.03, 0.23) | 0.08 (-0.02, 0.18) | -0.07 (-0.20, 0.05) |
| Excluding whole grains | 0.68 (0.17, 1.19) | 0.08 (-0.03, 0.19) | 0.18 (0.08, 0.28) | 0.14 (0.03, 0.25) | 0.00 (-0.12, 0.13) |
| Excluding legumes | 0.34 (-0.15, 0.83) | 0.08 (-0.03, 0.18) | 0.11 (0.01, 0.20) | 0.08 (-0.02, 0.18) | -0.06 (-0.18, 0.06) |
| Excluding nuts | 0.62 (0.13, 1.11) | 0.11 (0.00, 0.21) | 0.15 (0.05, 0.25) | 0.11 (0.01, 0.22) | 0.00 (-0.12, 0.13) |
| Excluding dairy | 0.57 (0.09, 1.05) | 0.10 (0.00, 0.21) | 0.14 (0.05, 0.24) | 0.14 (0.04, 0.24) | -0.04 (-0.15, 0.08) |
| Excluding fish | 0.50 (0.01, 0.99) | 0.10 (0.00, 0.21) | 0.14 (0.05, 0.24) | 0.12 (0.02, 0.22) | -0.06 (-0.19, 0.06) |
| Excluding tea | 0.63 (0.14, 1.12) | 0.12 (0.02, 0.23) | 0.16 (0.06, 0.25) | 0.14 (0.04, 0.24) | -0.03 (-0.16, 0.09) |
| Excluding grain quality | 0.81 (0.31, 1.31) | 0.12 (0.01, 0.22) | 0.18 (0.08, 0.28) | 0.18 (0.07, 0.28) | 0.01 (-0.11, 0.13) |
| Excluding soft fats and oils | 0.66 (0.18, 1.14) | 0.13 (0.02, 0.23) | 0.16 (0.06, 0.25) | 0.14 (0.04, 0.24) | -0.02 (-0.14, 0.10) |
| Excluding red meat | 0.78 (0.29, 1.27) | 0.12 (0.01, 0.22) | 0.18 (0.08, 0.27) | 0.16 (0.06, 0.26) | 0.02 (-0.10, 0.14) |
| Excluding sugar-containing beverages | 0.54 (0.05, 1.02) | 0.12 (0.01, 0.22) | 0.14 (0.05, 0.24) | 0.12 (0.02, 0.23) | -0.05 (-0.17, 0.07) |
| Excluding alcohol | 0.65 (0.16, 1.14) | 0.14 (0.03, 0.24) | 0.15 (0.05, 0.25) | 0.14 (0.03, 0.24) | -0.03 (-0.16, 0.09) |
| Excluding salt | 0.58 (0.11, 1.05) | 0.11 (0.01, 0.21) | 0.15 (0.06, 0.24) | 0.13 (0.03, 0.23) | -0.03 (-0.15, 0.08) |
| Excluding folic acid | 0.48 (-0.02, 0.97) | 0.10 (-0.01, 0.21) | 0.15 (0.05, 0.24) | 0.14 (0.03, 0.24) | -0.08 (-0.20, 0.04) |

^1^Effect estimates are regression coefficients (β) for child IQ at 14 years with their 95%-confidence intervals (95%CIs), per 1-unit higher score on the diet quality during pregnancy by excluding one of 15 foods at a time and additionally adjusting for the excluded food group. Model adjusted for child sex and age when brain imaging was assessed, age of mother, maternal education, maternal ethnic background, and household income, smoking during pregnancy, maternal psychopathological symptoms during pregnancy, and mother’s energy intake, based on pooled results of imputed data.

Supplemental Figure 1. Study population flowchart

Mothers who completed the FFQs with valid data in the first trimester of gestation

**N = 6485**

Children with informed consent for MRI scan

- at age 10 years: **N = 2780**
- at age 14 years: **N = 2721**

- Children who did not participated phase 3, focus 9 years study (n = 1357)

- Children who did not visit neuroimaging research center or did not provide consent for MRI scan

(n = 2348)

Study population for analysis of diet quality during pregnancy and brain volumetric measures

- Sample with data at age 10 years: **N = 2223**
- Sample with data at age 14 years: **N = 1582**
- Sample with data at both ages: **N = 872**

Excluded:

- No structural MRI data (n = 33)

- With braces (n = 65)

- No/poor reconstruction on FreeSurfer (n = 421)

- Major incidental findings (n = 12)

- No gyrification data (n = 26)

- Children who did not participated phase 4, focus 13 years study (n = 1663)

- Children who did not visit neuroimaging research center or did not provide consent for MRI scan

(n = 2101)

Excluded:

- No structural MRI data (n = 80)

- With braces (n = 764)

- No/poor reconstruction on FreeSurfer (n = 276)

- Major incidental findings (n = 9)

- No gyrification data (n = 10)

Age 14 years

Age 10 years
